# Phenotypic characteristics of isolates of *Aspergillus *section *Fumigati *from different geographic origins and their relationships with genotypic characteristics

**DOI:** 10.1186/1471-2334-11-116

**Published:** 2011-05-09

**Authors:** María Guadalupe Frías-De León, Monserrat Zavala-Ramírez, Susana Córdoba, Gerardo Zúñiga, Esperanza Duarte-Escalante, Armando Pérez-Torres, Armando Zepeda-Rodríguez, Irma López-Martínez, María José Buitrago, María del Rocío Reyes-Montes

**Affiliations:** 1 Laboratorio de Micología Molecular Departamento de Microbiología y Parasitología, Facultad de Medicina, Universidad Nacional Autónoma de México (UNAM), Ciudad Universitaria No. 3000, México D.F., 04360, México; 2 Departamento de Micologia, INEI ANLIS "Dr. Carlos G. Malbrán", Av. Velez Sarsfield 563, 1281 Buenos Aires, Argentina; 3 Departamento de Zoología, Escuela Nacional de Ciencias Biológicas, Instituto Politécnico Nacional, México D.F., 11340, México; 4 Departamento de Biología Celular y Tisular, Facultad de Medicina, Universidad Nacional Autónoma de México (UNAM), Ciudad Universitaria No. 3000, México D.F., 04360, México; 5 Servicio de Micología, Centro Nacional de Microbiología, Instituto de Salud Carlos III, Madrid, España

## Abstract

**Background:**

Epidemiological studies worldwide have shown that *A. fumigatus *exhibits important phenotypic and genotypic diversity, and these findings have been of great importance in improving the diagnosis and treatment of diseases caused by this fungus. However, few studies have been carried out related to the epidemiology of this fungus in Latin America. This study´s aim is to report on the epidemiology of the fungus by analyzing the phenotypic variability of *Aspergillus *section *Fumigati *isolates from different Latin American countries and the relationship between this variability, the geographical origin and genotypic characteristics.

**Methods:**

We analyzed the phenotypic characteristics (macro- and micromorphology, conidial size, vesicles size, antifungal susceptibility and thermotolerance at 28, 37 and 48°C) of *A*. section *Fumigati *isolates from Mexico (MX), Argentina (AR), Peru (PE) and France (FR). The results were analyzed using analysis of variance (ANOVA) and Tukey's multiple comparison test to detect significant differences. Two dendrograms among isolates were obtained with UPGMA using the Euclidean distance index. One was drawn for phenotypic data, and the other for phenotypic and genotypic data. A PCoA was done for shown isolates in a space of reduced dimensionality. In order to determine the degree of association between the phenotypic and genotypic characteristics AFLP, we calculated the correlation between parwise Euclidean distance matrices of both data sets with the nonparametric Mantel test.

**Results:**

No variability was found in the macromorphology of the studied isolates; however, the micromorphology and growth rate showed that the PE isolates grew at a faster rate and exhibited the widest vesicles in comparison to the isolates from MX, AR and FR. The dendrogram constructed with phenotypic data showed three distinct groups. The group I and II were formed with isolates from PE and FR, respectively, while group III was formed with isolates from MX and AR. The dendrogram with phenotypic and genotypic data showed the same cluster, except for an isolate from FR that formed a separate cluster. This cluster was confirmed using PCoA. The correlation between the phenotypic and genotypic data of the isolates revealed a statistically significant association between these characteristics.

**Conclusions:**

The PE isolates showed specific phenotypic characteristics that clearly differentiate them from the rest of the isolates, which matches the genotypic data. The correlation between the phenotypic and genotypic characteristics showed a statistically significant association. In conclusion, phenotypic and genotypic methods together increase the power of correlation between isolates.

## Background

*Aspergillus fumigatus Fresenius *is a filamentous, saprophytic fungus of great biological importance, as it is one of the major opportunistic pathogens causing invasive aspergillosis (IA) in immunosuppressed patients [1,2]. Its association with this type of patients depends on the host characteristics and on some typical phenotypic features that contribute to its pathogenicity such as its nutritional versatility, growth rate and efficient sporulation at a temperature of 37°C or higher [3,4].

*Aspergillus *section *Fumigati *has recently been reclassified by Samson et al. [5]. It currently contains 25 different species, with 8 anamorphs and 17 telemorphs. In the section *Fumigati*, besides *A. fumigatus*, other species, such as *Neorsartorya fischeri, N. pseudofischeri, N. hiratsukae, and A. lentulus*, have been reported to be human pathogens [6-8].

There are few studies available on the phenotypic characterization of *A. fumigatus*. The most relevant have reported variability in the pigment and texture of the colonies, growth rate at different temperatures, size and shape of the conidia, atypical phialides and differences in the size and shape of the conidial heads [9-13].

Other studies have explored the utility of phenotypic attributes (serological, immunochemical analysis of isozymes and protein profile) and genotypic characteristics (RFLP [Restriction Fragment Length Polymorphism], RAPD [Random Amplification Polymorphic DNA]-PCR and partial sequences of DNA) of *A. fumigatus *isolates for classification [10,11,14-19]. Some studies have also tried to find correlations between the phenotypic and genotypic characteristics, but these studies have so far shown no strict correlation between the compared attributes [11,12]. However, it is very important to consider certain phenotypic characteristics, as they are useful from an epidemiological point of view and can exhibit differences both within the same species and between different species. In patients with different clinical forms of aspergillosis, the *A. fumigatus *species has shown differences in its phenotypic characteristics. Depending on the clinical form and the duration of the illness, these morphological changes appear to be the result of selective pressure that is exerted by the microenvironment to which each organism is exposed to [10]. Differences in the phenotypic characteristics have also helped to identify new species, as in the case of *A. lentulus*, which showed differences in the rate of sporulation and conidial head size and low antifungal susceptibility [7]. Furthermore, Mesa-Arango et al. [20] observed morphological differences in isolates of *Sporothrix schenckii *that correlated with the geographic region and a special clinical form. Given these findings, in this study the phenotypic variation was analyzed in isolates of *A*. section *Fumigati *from Mexico (MX), Argentina (AR), Peru (PE) and France (FR) to find associations between the phenotypic characteristics (the micro- and macromorphology, size of conidia, size vesicles, thermotolerance and susceptibility to antifungal) and the geographic origin of these isolates, as well as to determine the correlation between these traits and previously reported genotypes that were generated by AFLP (Amplified Fragment Length Polymorphism) [21].

## Methods

### Fungal isolates

Fifty-five isolates of *A*. section *Fumigati *were used from different sources and geographical origins. The *A*. section *Fumigati *isolates studied were from patients with IA and aspergilloma from AR (n = 24), MX (n = 13), FR (n = 9), and PE (n = 4); environmental isolates were from AR (n = 3) and MX (n = 2). Data for each isolate are shown in Table 1. The fungal isolates were grown on potato dextrose agar (PDA) (Bioxon^®^, Mexico City, MX) at 37°C. The fungal isolates were identified according to the following criteria: development of the conventional colonial morphology, the presence of typical microscopic conidia, and their ability to grow at 48°C.

**Table 1:** *A*. section *Fumigati *isolates studied and their phenotypic characteristics

Isolate	Source	Origin	Conidia size (μm)	Vesicle size (μm)	%GI		Thermotolerance			MIC (mg/L)		
							
					28°C	48°C	28°C	37°C	48°C	ITZ	VCZ	AMB
AmbIII	E	AR	2.743	17.4	47	3.9	TS	TR	TR	0.13	0.13	0.25
AmbV	E	AR	2.564	17.9	33.9	6.8	TR	TR	TR	0.13	0.13	0.25
AmbVIII	E	AR	2.743	17.6	38.6	10.5	TS	TR	TR	0.13	0.13	0.25
951740	C^a^	AR	2.743	17.9	28.3	6	TR	TR	TR	0.13	0.25	1
951744	C^a^	AR	2.743	17.9	19.6	15	TR	TR	TS	0.25	0.13	0.5
951746	C^a^	AR	2.743	17.9	28.6	6.1	TR	TR	TR	0.25	0.13	1
951722	C^ND^	AR	2.743	17.1	41.2	7.8	TS	TR	TR	0.25	0.25	1
951733	C^a^	AR	2.743	17.6	36.8	12.3	TS	TR	TR	0.25	0.25	0.5
951734	C^a^	AR	2.743	17.9	13.6	9.1	TR	TR	TR	0.25	0.25	1
951736	C^a^	AR	2.743	17.6	27.1	30.5	TR	TR	TS	0.25	0.25	2
951737	C^a^	AR	2.743	17.9	55	11.7	TS	TR	TR	0.25	0.25	2
951738	C^a^	AR	2.743	16.9	48.4	51.6	TS	TR	TS	0.5	0.25	2
951739	C^a^	AR	2.743	17.9	48.7	33.3	TS	TR	TS	0.25	0.5	1
951740a	C^a^	AR	2.743	17.9	25	11.4	TR	TR	TR	0.13	0.25	0.25
951741	C^a^	AR	2.743	17.9	44.9	22.5	TS	TR	TS	0.25	0.13	0.5
951744a	C^a^	AR	2.743	17.9	29.1	9.1	TR	TR	TR	0.13	0.13	0.5
951745	C^a^	AR	2.743	17.9	36.2	12.1	TS	TR	TR	0.25	0.25	2
951746a	C^a^	AR	2.743	17.9	45.8	8.6	TS	TR	TS	0.06	0.13	0.5
951747	C^a^	AR	2.743	17.6	38	8	TS	TR	TR	0.25	0.25	2
982928	C^ND^	AR	2.564	17.6	40.4	8.8	TS	TR	TR	0.13	0.25	1
993315	C^c^	AR	2.564	16.6	42.3	1.9	TS	TR	TR	0.25	0.13	1
9272	C^d^	AR	2.564	17.4	46.1	7.7	TS	TR	TR	> 16	0.25	0.5
8571	C^d^	AR	2.564	17.1	36.2	13.8	TS	TR	TR	> 16	0.25	1
6578	C^b^	AR	2.564	17.1	41.3	18.9	TS	TR	TS	> 16	0.25	1
MM-7	E	MX	2.564	17.9	42.5	40	TS	TR	TS	0.25	0.25	1
MM-8	E	MX	2.564	17.6	36.8	39.5	TS	TR	TS	0.03	0.25	0.25
MM-9	C^c^	MX	2.64	17.6	39.6	8.6	TS	TR	TR	0.5	0.25	1
MM-10	C^c^	MX	2.564	18.1	50.8	22.2	TS	TR	TS	0.25	0.25	1
MM-11	C^c^	MX	2.564	17.9	45.2	12.9	TS	TR	TS	0.5	0.13	1
MM-32	C^c^	MX	2.897	17.9	52.9	15.7	TS	TR	TS	0.06	0.13	0.25
MM-33	C^c^	MX	2.564	18.1	48.3	6.7	TS	TR	TR	0.13	0.25	0.5
MM-34	C^c^	MX	2.564	18.1	45.8	8.5	TS	TR	TR	0.13	0.25	1
MM-35	C^c^	MX	2.82	17.9	43.6	25.4	TS	TR	TS	0.13	0.25	0.5
MM-36	C^c^	MX	2.564	18.1	38	11.3	TS	TR	TR	0.13	0.25	0.5
MM-37	C^c^	MX	2.564	18.1	38.6	50	TS	TR	TS	0.13	0.5	0.5
MM-38	C^c^	MX	2.64	17.6	40	7.3	TS	TR	TR	0.13	0.25	1
MM-39	C^c^	MX	2.564	18.4	43.3	10	TS	TR	TR	0.06	0.25	0.5
MM-45	C^c^	MX	2.564	17.9	35.8	7.5	TS	TR	TR	0.03	0.06	0.5
MM-46	C^c^	MX	2.564	17.9	36.8	10.5	TS	TR	TR	0.13	0.13	0.25
Af-8	C^b^	FR	2.564	20.7	36.8	1.8	TS	TR	TR	0.1	0.25	0.5
Af-11	C^b^	FR	2.564	20.7	37.3	25.4	TS	TR	TS	0.03	0.25	0.5
Af-15	C^b^	FR	2.564	21	41.9	24.2	TS	TR	TS	0.03	0.25	1
Af-22	C^b^	FR	2.564	21	37.5	12.5	TS	TR	TR	0.03	0.13	0.25
Af-26	C^b^	FR	2.564	20.5	45.9	11.5	TS	TR	TR	0.03	0.25	0.5
Af-29	C^b^	FR	2.743	20.5	31.4	5.9	TR	TR	TR	0.06	0.25	0.5
Af-34	C^b^	FR	2.564	20.7	32.1	5.7	TR	TR	TR	0.03	0.13	0.25
Af-35	C^b^	FR	2.64	20.5	48.5	19.7	TS	TR	TS	0.06	0.25	0.5
Af-41	C^b^	FR	2.564	20.7	32.1	7.5	TR	TR	TR	0.03	0.13	0.5
51435	C^b^	PE	2.743	23.3	13.6	10.6	TR	TR	TR	0.13	0.13	0.5
51594	C^d^	PE	2.564	23.1	5.9	1.5	TR	TR	TR	0.13	0.13	0.5
53027	C^b^	PE	2.64	23.3	14.3	7.1	TR	TR	TR	0.13	0.13	0.5
53097	C^b^	PE	2.564	23.6	15.2	9.1	TR	TR	TR	0.13	0.25	0.5

* The mean of the diameters of 30 conidia; ^+ ^The mean of the widths of 10 vesicles; TS- thermosensitive; TR- heat-resistant; C- clinical source; E- environmental source; AR- Argentina; MX- Mexico; FR- France; PE- Peru; ^a ^bronchial secretion, ^b ^sputum, ^c ^bronchoalveolar lavage, ^d ^lung biopsy, ^e ^paranasal biopsy, ^ND ^undeterminated.

### Monospore cultures

From each isolate grown on PDA (Bioxon^®^) for 2-4 d at 37°C, a conidial suspension was prepared with 1 mL of phosphate buffer (pH 7.4) and 0.05% Tween 20 (PBST). This suspension was diluted (1:1000) and 50 μL was used for growth on PDA medium (Bioxon^®^). The Petri dishes were incubated at 37°C and observed for colonial growth. Only one colony was selected from each plate, and grown in PDA (Bioxon^®^) agar slants at 37°C. The conidia of the monospores were conserved in sterile water at 4°C.

### Macromorphology

The isolates of *A*. section *Fumigati *grown on PDA (Bioxon^®^) at 37°C for 4 d were observed to identify the morphological characteristics of each culture (the color and texture of the colony).

### Micromorphology

The micromorphological characteristics of the isolates of *A*. section *Fumigati *were analyzed using the microculture method of Ridell [22]. The microculture was incubated at 37°C for 4 d or until the fungus was observed to grow. Subsequently, the cover slip was carefully separated from the agar, placed on a slide with a drop of cotton blue stain and observed under the microscope. We analyzed the morphological characteristics of the conidia and the size of the vesicles. The width of 10 vesicles was measured with a calibrated ocular micrometer (Olympus America Inc., Melvilla, NY, USA).

### Thermotolerance

Thermotolerance was measured as described by Mesa-Arango et al. [20]. A suspension of 5 × 10^3 ^conidia/mL was prepared with PBST using a Neubauer chamber and inoculated it in triplicate for each temperature (28, 37 and 48°C) considering the growth of each isolate on PDA (Bioxon^®^) for 4 d at 37°C. We later determined the growth rate of the isolates at each temperature using the following formula: [(colony diameter - inoculum diameter)/days of incubation]. We also calculated the percentage of growth inhibition (%GI) at 28 and 48°C using the following formula: [(colony diameter at 37°C - colony diameter at 28 or 48°C)/colony diameter at 37°C] X 100. To identify heat-sensitive or heat-resistant isolates of *A*. section *Fumigati*, we obtained the average %GI at 28 and 48°C by considering all 55 isolates. All isolates that showed a %GI higher than average were considered heat-sensitive, while those with a smaller value of %GI, less than the average, were classified as heat-resistant.

### Conidia size

The diameter of 30 conidia for each *A*. section *Fumigati *isolate grown on PDA (Bioxon^®^) and incubated at 37°C for 4 d, was measured with a calibrated ocular micrometer (Olympus America Inc.).

### Scanning electron microscopy (SEM)

Samples obtained from microcultures of fungal isolates selected from each country were processed using a modification of the standard procedures for SEM examination [23,24]. Specimens were fixed on a mixture of 4% paraformaldehyde and 0.5% glutaraldehyde in sodium cacodylate buffer (0.2 M, pH 7.2) for 5 h at 4°C. After three washes of 10 min each with the same cacodylate buffer, the microcultures were postfixed with osmium tetroxide at 1% for 2 h. We proceeded to manually cut the excess agar from both the base and the ends of the samples. The specimens were washed as mentioned and were then dehydrated with increasingly graded ethanol, from 30% to absolute ethanol, at room temperature. Soon after, the samples were dried by the critical point method in a Samdri 780A desiccator (Rockville, MD, USA) using CO_2_. The samples were mounted on aluminium cylinders with silver paste and placed in a high vacuum evaporator for gold coating for 6 min in a metal ionizing JEOL JFC-1100 (Fine Coat^® ^ion Sputter, JEOL Ltd, Tokyo, JP). The samples were analyzed with a Zeiss DSM-950 scanning electron microscope (Carl Zeiss, Jena, DE) at 25 kV and a 10 mm working distance.

### Antifungal susceptibility

The monospore cultures were tested for antifungal susceptibility according to the guidelines in the M38-A2 document of the CLSI (Clinical and Laboratory Standards Institute) [25]. For this test, we used amphotericin B (AMB) (Sigma Chemical Co., St. Louis Mo, USA), itraconazole (ITZ) (Janssen Pharmaceutical S.A., Buenos Aires, AR) and voriconazole (VCZ) (Pfizer S.A., Buenos Aires, AR) as standard powders of known potency. The stock solutions of each antifungal agent were prepared with dimethyl sulfoxide (Sigma) and diluted with RPMI 1640. Concentration used ranged from 0.0313 to 16 mg /L for the three antifungals. The drug dilutions were placed on 96-well microdilution plates (Nunclon 167008, Nunc, Naperville, IL, USA). The microplates were incubated without agitation at 35°C for 46-50 h. Although there are no defined cutoff values for these antifungal agents, we considered a minimum inhibitory concentration (MIC) value ≥ 4 mg/L as indicating resistance to the drug.

### Data analysis

An analysis of variance (ANOVA) (α = 0.01 and 0.05) and Tukey's multiple comparison test were used to detect significant differences in the conidia size and growth rate at different temperatures. The analyses were performed using SPSS (Statistical Package for the Social Sciences) version 12.0 [26].

The thermotolerance results were tested at three temperatures: 28, 37 and 48°C and based on the rate of growth of the colonies (1-7 cm diameter), six categories for each temperature were defined as follows: I (1.0-2.0 cm), II (2.1-3.0 cm), III (3.1-4.0 cm), IV (4.1-5.0 cm) V (5.1-6.0 cm) and VI (6.1-7.0 cm). These categories were coded as multistate character without logical sequence, and together with characters of the conidia and vesicles size were integrated in a single matrix. Pairwise distances among isolates were estimated with Euclidean distance index [27] and a dendrogram was constructed by the Unweighted Pair Group Method with Arithmetic Averages (UPGMA). However, to obtain a best relationship among isolates, we also integrated in a single matrix the phenotypic data obtained in this study and genotypic data (AFLP) reported by Duarte-Escalante et al. [21] of the same isolates (data not shown). A dendrogram of this data set was constructed by UPGMA. The reliability of both dendrograms was evaluated by means of the cophenetic correlation coefficient using the Mantel test [28] after 10,000 permutations and bootstrap. In addition, we carried out a Principal Coordinate Analysis (PCoA) with pairwise Euclidean distances of isolates derived of phenotypic-genotypic data matrix. This method facilitates the detection of associations more complex among isolates in a multidimensional scenario. Finally, a correlation between pairwise Euclidean distance matrices of phenotypic and genotypic characteristics was estimated with Mantel´s non-parametric test [28] after 10,000 permutations. All these analyses were carried out with NTSYS-PC v 2.0 [29].

## Results

### Macromorphology

All isolates had the typical macromorphology of the species. The color of the front was teal with a white border, and the back was cream-colored. The texture of the colonies was velvety or powdery.

### Micromorphology

Micromorphology showed different sizes (widths) of vesicles. The PE isolates showed the widest vesicles (23.35 ± 0.75 μm), followed by FR (20.73 ± 0.83 μm), MX (17.97 ± 0.84 μm) and AR (17.62 ± 0.78 μm). However, these sizes are within the range that is described for *A. fumigatus*.

### Thermotolerance

All of the isolates grew at the three tested temperatures, with 37°C being the optimal temperature for growth (Table 1). However, statistically significant differences were found (P < 0.05) in the growth rates of the isolates at the three temperatures used. The isolates from PE grew faster than did those from FR, MX and AR (Table 2).

**Table 2:** Data analysis of the phenotypic characteristics of isolates of *A*. section *Fumigati*

Origin	Conidia size (μm) ± SD	Growth rate (mm/ day) ± SD			Vesicles size (μm) ± SD
			
		28°C	37°C	48°C	
MX	2.61 ± 0.35^ab^	3.21 ± 0.37^b^	5.60 ± 1.00^b^	4.69 ± 1.31^b^	17.97 ± 0.84^d^
PE	2.62 ± 0.40^ab^	5.91 ± 0.37^a^	6.74 ± 0.29^a^	6.77 ± 0.50^a^	23.35 ± 0.75^a^
FR	2.58 ± 0.22^b^	3.55 ± 0.19^b^	5.74 ± 0.54^b^	5.15 ± 0.53^b^	20.73 ± 0.83^c^
AR	2.72 ± 0.62^a^	3.34 ± 0.56^b^	5.32 ± 0.66^b^	4.51 ± 0.81^b^	17.62 ± 0.78^b^

α = 0.05; *SD- Standard Deviation; **Same letters represent no significant differences (P < 0.05) between the countries; a, b, c, d = Groups. AR- Argentina; MX- Mexico; FR- France; PE- Peru.

According to the values obtained for %GI (Table 1), there were differences in thermotolerance. At 28°C, the thermosensitive isolates were those with a %GI ≥ 36, where the heat-resistant isolates had a value of < 36. At 48°C, the thermosensitive isolates were defined as having a %GI ≥ 14, and the heat-resistant isolates had values of < 14. At 28°C, 100% of the isolates from PE were heat-resistant, while only 52%, 33% and 7% of the isolates from AR, FR and MX, respectively, were heat-resistant. At 48°C, all of the isolates from PE were also heat-resistant, and 70, 60 and 67% of the isolates from AR, MX and FR, respectively, were also heat-resistant at this temperature (Table 1).

### Conidia size

The diameters of the conidia of all isolates ranged from 2.56 to 2.89 μm. The conidia of the isolates from AR were the largest (2.72 ± 0.62), followed by those from PE (2.62 ± 0.40), MX (2.61 ± 0.35) and FR (2.58 ± 0.22). The average diameter of the conidia for the isolates from AR and FR were significantly different (P < 0.05) (Table 2).

### Scanning electron microscopy

**Figure 1:**
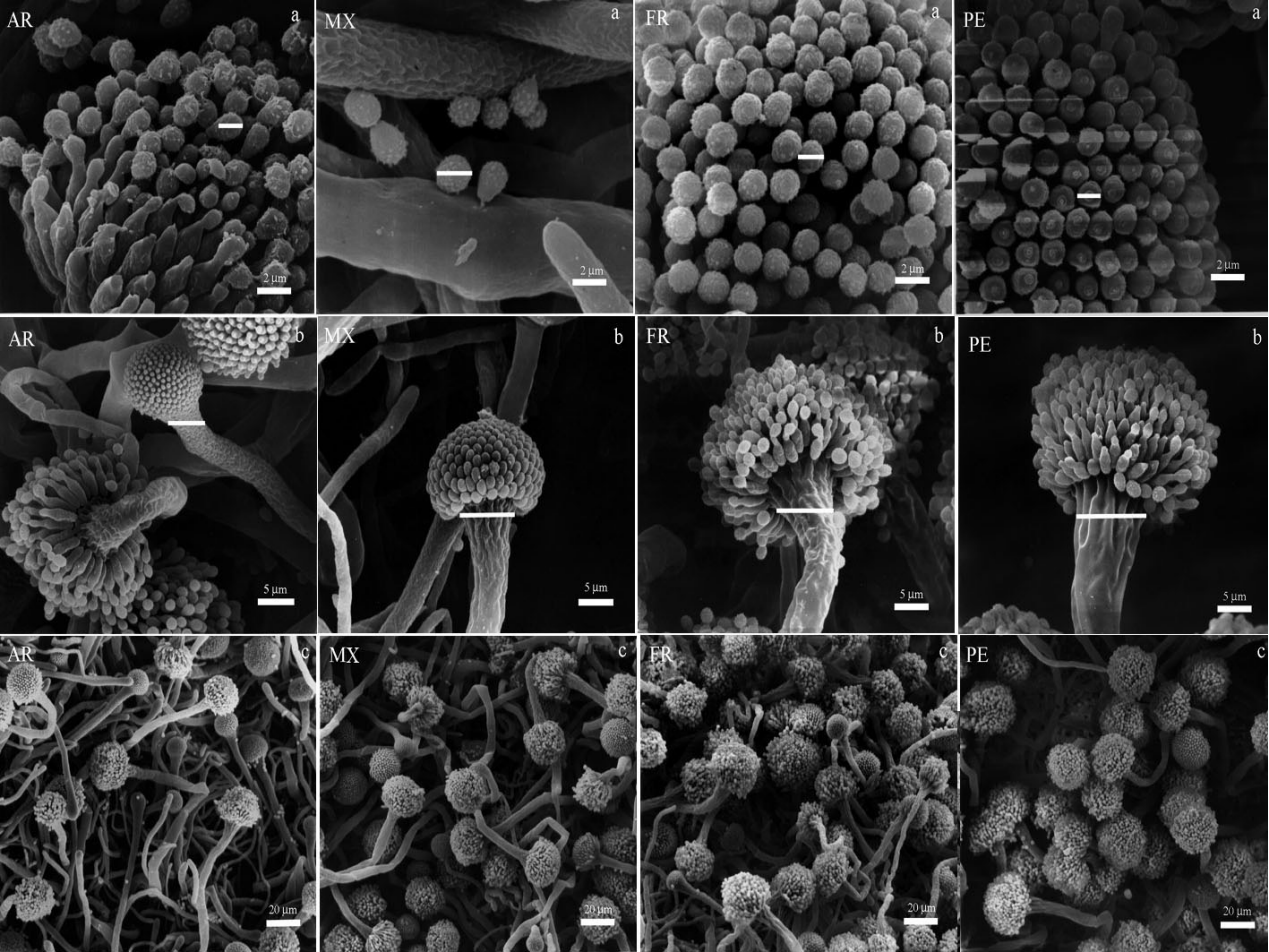
**Scanning Electron Microscopy of the phenotypic characteristics of *A*. section *Fumigati *isolates selected from each country**. Conidia size (a); vesicles size (b) and growth rate (c). The samples were processed with a conventional technique for SEM. Abbreviations: AR-Argentina; MX-Mexico; FR- France and PE-Peru.

SEM analysis confirmed the statistically significant differences that were observed in the photon microscopy analysis on the size of the conidia and the vesicles of the *A*. section *Fumigati *isolates selected from each country (Figure 1). The conidia of the isolates from AR were the largest, followed by those from PE, MX and FR. In reference to the size of the vesicles of the isolates selected from each country, we observed that the PE isolates had widest vesicles, followed by FR, MX and AR. Similarly, there was greater sporulation in PE isolates proven by the presence of conidial heads while isolates from MX, AR and FR had less conidial heads (Figure 1c).

### Antifungal Susceptibility

With regard to antifungal susceptibility drugs, the range of the MICs for VCZ for all isolates was 0.06 to 0.5 mg/L, for ITZ 0.03 to ≥ 16 mg/L and for AMB 0.25 to 2.0 mg/L. The antifungal susceptibility results varied (Table 1), and there were no isolates that were resistant to AMB and VCZ only to ITZ. The AR isolates (9272, 8571, 6578) were resistant to ITZ. Isolates from MX, FR and PE showed no resistance to antifungal drugs (Table 1).

### Data Analysis

**Figure 2:**
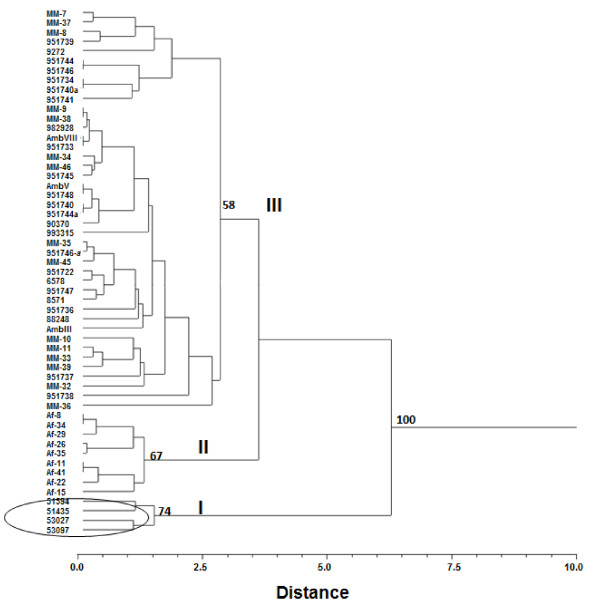
**Dendrogram based on phenotypic data (conidia and vesicle size and thermotolerance), generated by UPGMA using Euclidean distances (*r *= 0.89859, *P *= 0.0004) of *A*. section *Fumigati *isolates from MX, AR, FR and PE**. The number on the nodes represents the support values based on 10,000 bootstrap replicas.

The dendrogram obtained with phenotypic data of the *A*. section *Fumigati *isolates, yielded three clusters (Figure 2). The first cluster (group I) included all the isolates from PE with a high association between them and a bootstrap of 74%. The second cluster (group II) consisted of isolates from FR, whose bootstrap was of 67%. Group III consisted of isolates from MX and AR with major distances and a bootstrap of 58%.

**Figure 3:**
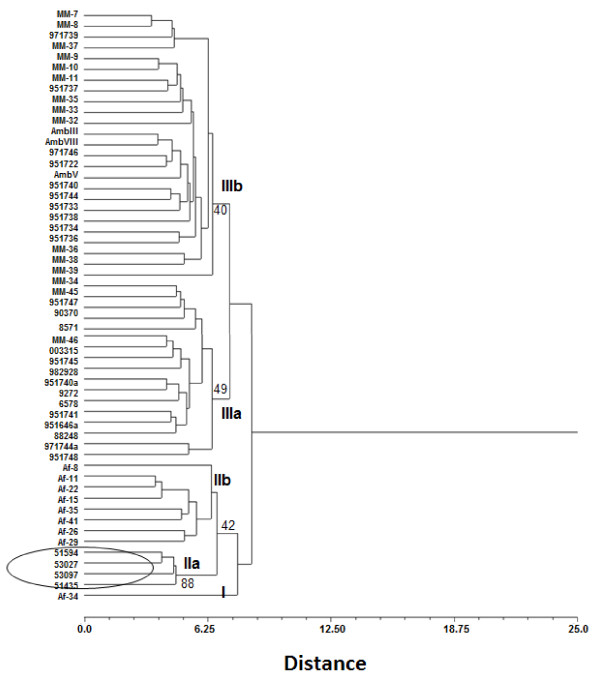
**Dendrogram based on phenotypic (conidia and vesicle size and thermotolerance) and genotypic data (AFLP), generated by UPGMA using Euclidean distances (*r *= 0.78763, *P *= 0.0004) of 55 *A*. section *Fumigati *isolates from MX, AR, FR and PE**. The number on the nodes represents the support values based on 10,000 bootstrap replicas.

The dendrogram generated with the similarity matrices of pheno and genotypic data of the *A*. section *Fumigati *isolates yielded three clusters (Figure 3). The first cluster (group I) consisted only one isolate from FR relatedness with the group II. The second cluster (group II) showed two subgroups. Subgroup IIa included all isolates from PE, and subgroup IIb included isolates from FR. Finally, the third cluster (group III) also contained two subgroups well differenced. Subgroup IIIa consisted of isolates from MX and AR. Subgroup IIIb consisted of isolates from MX and AR. Bootstrap values for into this groups were low but among them were above from 50%.

**Figure 4:**
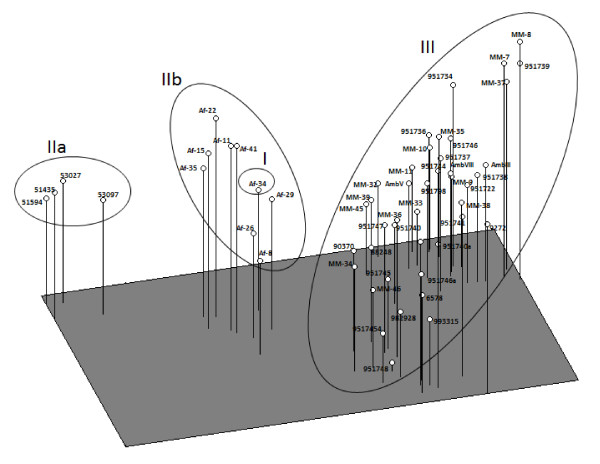
**Principal coordinates analysis (PCoA) of pheno and genotypic data of 55 *A*. section *Fumigati *isolates from MX, AR, FR y PE in a multidimensional space**.

These results are reinforced by the PCoA (Figure 4) that shows three groups that depended almost on the geographical origins of the isolates (MX, AR, FR and PE). The first three principal coordenates of the analysis explained 51.65% of the observed variation, specifically the first component explained 28.66% of variation, the second component 15.50%, and the third component 6.49%. These findings largely correspond to the clustering obtained by UPGMA (Figure 3).

When we correlated the pairwise Euclidean distance matrices of the phenotypic characteristics (size of the conidia, size of the vesicles and thermotolerance) with the genotypic characteristics (AFLP) of the studied *A*. section *Fumigati *isolates, a statistically significant relationship (r = 0.105, p = 0.038) was found, showing there is an association between the two types of characteristics.

## Discussion

The *A. fumigatus *fungus is widely distributed around the world. In Latin American countries, not much research has been done to establish their phenotypic and genotypic variability. To provide more information regarding this fungus, we studied the phenotypic characteristics (the micro- and macromorphology, size of the conidia, vesicles size, thermotolerance and antifungal susceptibility) of clinical and environmental isolates of *A. fumigatus *from MX, AR, PE and FR, and we assessed the relationship between the phenotypic and the genotypic characteristics reported in the literature for these isolates [21], to determine whether the variability of the phenotypic characteristics correlated with the genotypic characteristics. The phenotypic characterization was not different from that reported for the specie; however, some interesting observations are worth highlighting concerning the size of the vesicles, thermotolerance and growth rate at different temperatures (28, 37 and 48°C). The PE and AR isolates were characterized by exhibiting larger conidia (Figure 1), although the size range found matches that reported by Rinyu et al. [11], who determined that the size of the conidia of clinical and environmental *A. fumigatus *isolates from different countries was 2.5-3.5 μm. The sizes of the vesicles varied according to their geographic origin, and the PE isolates had the widest vesicles (Figure 1). Another interesting phenotypic characteristic was the thermotolerance at 28, 37 and 48°C, where all isolates grew at the three temperatures; however, the PE isolates had the highest growth rate at 48°C (Table 2). The thermotolerance and growth rate have been determined to be virulence factors [3,4,30], and it is therefore possible that the isolates from PE, due to their phenotypic characteristics, are more virulent than are those from AR, MX and FR. In other fungi, it has also been observed that the virulent strains have specific phenotypic characteristics that differentiate them from those that are less virulent. In *H. capsulatum*, the dimorphic transition rate at 37°C was found to be a key factor in the pathogenicity of three strains with different degrees of virulence that were studied, as that with the highest virulence was transformed to the yeast phase (virulent) more quickly [31]. Phenotypic differences associated with geographical areas have also been observed in other human pathogens, such as *S. schenckii *isolates from MX, Colombia (CO) and Guatemala (GT). The isolates from CO were heat-resistant and had larger conidia than those from MX and GT. Furthermore, genotyping by RAPD-PCR showed that these isolates were grouped according to their geographical origin [20]. The authors also suggested that the characteristics of the isolates from CO were related to the clinical form of the disease, as the fixed form of sporotrichosis predominates in CO.

Because the isolates from PE showed characteristics that were different from the other isolates studied, we measured their antifungal susceptibility agents AMB, ITZ and VCZ. Some new species of *A*. section *Fumigati*, for example *A. lentulus*, show an association between resistance to antifungal and slow sporulation [32]. However, this study found no such association in the group from PE, with only the AR fungus showing resistant isolates. In this study, resistance to antifungal showed no association to their geographical origin.

However, the dendrogram built based on the phenotypic characteristics (thermotolerance, conidia and vesicle sizes) (Figure 2) show that the isolates from FR and PE cluster according to their geographical origin, while isolates from MX and AR form a single cluster unrelated to their geographical origin. This is contrary to what is observed when analyzing the genotypical characteristics, where the majority of the isolates separate according to geographic origin [21]. This confirms that these molecular markers are more sensible to the discrimination between isolates by geographic origin. On the other hand, when the dendrogram was built including the phenotypes and genotypes (Figure 3), it was seen that the isolation of the Af-34 from FR formed an independent group not shown in the dendrogram built only with the phenotypic data, nor recorded in Duarte-Escalante et al. [21]. This cluster is confirmed using PCoA. It is interesting to note that the discrimination power increases when the phenotypic and genotypic methods are analyzed together.

It is noteworthy that although the phenotypic characteristics do not differentiate phylogenetic species of *A*. section *Fumigati*, they do help show the intraspecies differences [33], since the species included in this section have morphological differences which may lead to an erroneous identification and reason why these organisms are discarded since they are considered as contaminants. To avoid this error, it is important to take the morphological differences into consideration to later confirm the presence of another species using genotypical methods, such as the analysis of partial sequences of the ß-tubulin-encoding gene (*benA*), the mitochondrial cytochrome b-encoding gene (*mtcytb*), the rodlet A encoding gene (*rodA*), the salt-responsive gene, the internal transcribed spacer (ITS)1-5.8S-ITS2 region, the genes encoding calmodulin and actin [5,7,17,33-35]. Additionally, it is important to note that a good differentiation, whether intra or interspecies, is based on the results of the combination of phenotypic and genotypic methods since the phenotypic characteristics by themselves do not differentiate them but provide a hint towards the identification of a species later confirmed using molecular methods.

On the other hand, when distance matrices are generated from phenotypic and genotypic (AFLP) characteristics, we found a low correlation (r = 0.0105), probably due to the number of PE isolates studied. However, the association between them was statistically significant (p = 0.032). This association again supports that certain phenotypical characteristics (growth rate and vesicle size) work together to cluster the isolates according to geographical origin. However, it is noteworthy that the phenotypic characteristics may vary depending on the surrounding environmental conditions where the fungus grows since the expression of a phenotypic character is the result of the interaction of the genotype with the environment and is therefore likely to be modified when environmental conditions such as climate, age of culture or incubation temperature vary. In addition, it may be the case that the genetic information is not completely expressed and the phenotype reflects only a small part of this information [36].

## Conclusions

The correlation between the phenotypic and genotypic characteristics showed a statistically significant association. We consider that the phenotypic and genotypic methods together increase the power discrimination between isolates. Moreover, it is pertinent to point out that in order to obtain greater knowledge about the phenotypic variability of *A. fumigatus*, it is important to gather a greater number of isolates from PE and FR. It also highlights the need for each country to carry out specific studies for determining the molecular or phenotypic tools that are appropriate for the correct identification of the fungus and for providing proper treatment. These studies will also identify sources of infection, resulting in a better control of aspergillosis.

## Competing interests

The authors declare that they have no competing interests.

## Authors' contributions

MGFDL and MZR conducted the experiments. SC contributed isolates from Argentina and carried out the test of antifungal susceptibility agents. EDE and GZ participated in the statistical analysis of the study. APT, AZR and ILM performed the scanning electron microscopy analysis. MJB provided a critical review of the manuscript. MRRM designed the study and supervised the laboratory procedures and the data analysis. MGFDL and MRRM analyzed and interpreted the results and wrote the manuscript. All authors contributed to writing the manuscript and approved its final version.

## Pre-publication history

The pre-publication history for this paper can be accessed here:

http://www.biomedcentral.com/1471-2334/11/116/prepub

## References

[B1] Latgé JP *Aspergillus fumigatus *and aspergillosis Clin Microbiol Rev 1999 12 310 350 10194462 10.1128/cmr.12.2.310 PMC88920

[B2] Latgé JP *Aspergillus fumigatus*, a saprotrotophic pathogenic fungus Mycologist 2003 17 56 61 10.1017/S0269915X0300209X

[B3] Araujo R Rodrigues AG Variability of germinative potencial among pathogenic species of *Aspergillus* J Clin Microbiol 2004 42 4335 4337 10.1128/JCM.42.9.4335-4337.2004 15365039 PMC516339

[B4] Rhodes JC *Aspergillus fumigatus*: Growth and virulence Med Mycol 2006 44 S77 S81 10.1080/13693780600779419 17050423

[B5] Samson RA Hong S Peterson SW Frisvad JC Varga J Polyphasic taxonomy of *Aspergillus *section *Fumigati *and its teleomorph *Neosartorya* Stud Mycol 2007 59 147 203 10.3114/sim.2007.59.14 18490953 PMC2275200

[B6] Balajee SA Gribskov J Brandt M Ito J Fothergill A Marr KA Mistaken identity: *Neosartorya pseudofischeri *and its anamorph masquerading as *Aspergillus fumigatus* J Clin Microbiol 2005 43 5996 5999 10.1128/JCM.43.12.5996-5999.2005 16333088 PMC1317194

[B7] Balajee SA Gribskov JL Hanley E Nickle D Marr KA *Aspergillus lentulus *sp. nov., a new sibling species of *A. fumigatus* Eukaryot Cell 2005 4 625 632 10.1128/EC.4.3.625-632.2005 15755924 PMC1087803

[B8] Guarro J Kallas EG Godoy P Karenina A Gene J Stchigel A Colombo AL Cerebral aspergillosis caused by *Neosartorya hiratsukae*, Brazil Emerg Infect Dis 2002 8 989 991 12194781 10.3201/eid0809.020073 PMC2732550

[B9] Marsella R Mercantini R Stefanini A Volterra L An atypical isolate of *Aspergillus fumigatus *from a man affected with a pulmonary disease Mykosen 1983 26 201 206 6346091

[B10] Leslie CE Flannigan B Milne LJ Morphological studies on clinical isolates of *Aspergillus fumigatus* J Med Vet Mycol 1988 26 335 341 10.1080/02681218880000481 3073205

[B11] Rinyu E Varga J Ferenczy L Phenotypic and genotypic analysis of variability in *Aspergillus fumigatus* J Clin Microbiol 1995 35 2567 2575 10.1128/jcm.33.10.2567-2575.1995 PMC228530 8567884

[B12] Schmidt A Wolff MH Morphological characteristics of *Aspergillus fumigatus *strains isolated from patient samples Mycoses 1997 40 347 351 10.1111/j.1439-0507.1997.tb00248.x 9470420

[B13] Chanteperdrix V Bourgerette E Gantier JC de Faverges G Herman D Lauby M Mycological examination of a non-uniseriate *Fumigati *section's *Aspergillus* Ann Biol Clin 2008 66 581 583 Paris 10.1684/abc.2008.0272 18957351

[B14] Burnie JP Coke A Matthews RC Restriction endonuclease analysis of *Aspergillus fumigatus *DNA J Clin Pathol 1992 45 324 327 10.1136/jcp.45.4.324 1349614 PMC495273

[B15] Matsuda H Kohno S Maesaki S Yamada H Koga H Tamura M Kuraishi H Sugiyama J Application of ubiquinone systems and electrophoretic comparison of enzymes to identification of clinical isolates of *Aspergillus fumigatus *and several other species of *Aspergillus* J Clin Microbiol 1992 30 1999 2005 1500506 10.1128/jcm.30.8.1999-2005.1992 PMC265431

[B16] Debeapuis JP Sarfati J Chazalet V Latgé JP Genetic diversity among clinical and environmental isolates of *Aspergillus fumigatus* Infect Immun 1997 65 3080 3085 9234757 10.1128/iai.65.8.3080-3085.1997 PMC175434

[B17] Wang L Yokoyama K Miyaji M Nishimura K Mitochondrial cytochrome b gene analysis *Aspergillus fumigatus *and related species J Clin Microbiol 2000 38 1352 1358 10747106 10.1128/jcm.38.4.1352-1358.2000 PMC86444

[B18] Rath PM Phenotipic and genotypic characterization of reference strains of the genus *Aspergillus* Mycoses 2001 44 65 72 10.1046/j.1439-0507.2001.00620.x 11413925

[B19] Katz ME Dougall AM Weeks K Cheetham BF Multiple genetically distinct groups revealed among clinical isolates identified as atypical *Aspergillus fumigatus* J Clin Microbiol 2005 43 551 555 10.1128/JCM.43.2.551-555.2005 15695644 PMC548029

[B20] Mesa-Arango AC Reyes-Montes MR Pérez-Mejía A Navarro-Barranco H Souza V Zúñiga G Toriello C Phenotyping and genotyping of *Sporothrix schenckii *isolates according to geographic origin and clinical form of sporotrichosis J Clin Microbiol 2002 40 3004 3011 10.1128/JCM.40.8.3004-3011.2002 12149366 PMC120692

[B21] Duarte-Escalante E Zúñiga G Nava-Ramírez O Córdoba S Refojo N Arenas R Delhaes L Reyes-Montes MR Population structure and diversity of the pathogenic fungus *Aspergillus fumigatus *isolated from different sources and geographic origins Mem Inst Oswaldo Cruz 2009 104 427 433 10.1590/S0074-02762009000300005 19547867

[B22] Ridell R Permanent stained micological preparations obtained by slide culture Mycology 1950 42 265 270 10.2307/3755439

[B23] Nowell JA Parules JB Preparation of experimental tissue for scanning electron microscopy 1980 AMSO-HARE 6999593

[B24] Bacilio-Jimenez M Aguilar-Flores S del Valle MV Perez A Zepeda A Zenteno E Endophytic bacteria in rice seeds inhibit early colonization of roots by *Azospirillum brasilense* Soil Biol Biochem 2001 33 167 172 10.1016/S0038-0717(00)00126-7

[B25] CLSI Reference method for broth dilution antifungal susceptibility testing of filamentous fungi; approved standard-second edition. CLSI document M38-A2 2008 Clinical and Laboratory Standards Institute (formely NCCLS) 940 West Valley Road, Wayne, Pa

[B26] Montgomery DC Diseño y Análisis de Experimentos 1991 Grupo Editorial Iberoamericana

[B27] Legendre P Legendre L Numerical Ecology 1998 Elsevier, Amsterdam

[B28] Manly JF Randomization bootstrap and montecarlo Methods in Biology 1997 Chapman and Hall, London

[B29] Rohlf FJ Numerical taxonomy and multivariate analysis system 1998 Exeter Software Inc., New York

[B30] Bhabhra R Askew DS Thermotolerance and virulence of *Aspergillus fumigatus*: role of the fungal nucleolus Med Mycol 2005 43 S87 S93 16110798 10.1080/13693780400029486

[B31] Keath EJ Painter AA Kobayashi G Medoff G Variable expression of a yeast-phase-specific gene in *Histoplasma capsulatum *strains differing in thermotolerance and virulence Infect Immun 1989 57 1384 1390 2565289 10.1128/iai.57.5.1384-1390.1989 PMC313287

[B32] Balajee SA Weaver M Imhof A Gribskov J Marr KA *Aspergillus fumigatus *variant with decreased susceptibility to multiple antifungals Antimicrob Agents Chemother 2004 48 1197 1203 10.1128/AAC.48.4.1197-1203.2004 15047520 PMC375298

[B33] Yaguchi T Horie Y Tanaka R Matsuzawa T Ito J Nishimura K Molecular phylogenetics of multiple genes on *Aspergillus *section *Fumigati *isolated from clinical specimens in Japan Nippon Ishinkin Gakkai Zasshi 2007 48 37 46 10.3314/jjmm.48.37 17287721

[B34] Balajee SA Nickle D Varga J Marr KA Molecular studies reveal frequent misidentification of *Aspergillus fumigatus *by morphotyping Eukaryot Cell 2006 5 1705 1712 10.1128/EC.00162-06 17030996 PMC1595351

[B35] Hong SB Go SJ Shin HD Frisvad JC Samson RA Polyphasic taxonomy of *Aspergillus fumigatus *and related species Mycologia 2005 97 1316 1329 10.3852/mycologia.97.6.1316 16722222

[B36] Bertout S Renaud F Barton R Symoens F Burnod J Piens MA Lebeau B Viviani MA Chapuis F Bastide JM Grillot R Mallié The Europan Research Group on Biotype and Genotype of Aspergillus Genetic Polymorphism of *Aspergillus fumigatus *in clinical samples from patients with invasive aspergillosis: Investigation using multiple typing methods J Clin Microbiol 2001 39 1731 1737 10.1128/JCM.39.5.1731-1737.2001 11325982 PMC88017

